# Hemodynamic and Genetic Associations with the Risk of Idiopathic Pulmonary Arterial Hypertension Development in an Ethnic Cohort of Kazakhs

**DOI:** 10.3390/diagnostics14232687

**Published:** 2024-11-28

**Authors:** Dana Taizhanova, Togzhan Nurpissova, Gulshara Abildinova, Tamilla Martynyuk, Nazgul Kulmyrzayeva, Elena Zholdybayeva

**Affiliations:** 1 Department of Internal Diseases, Karaganda Medical University Non-Commercial Joint Stock Company, Karaganda 100000, Kazakhstan; taizhanova_kgma@mail.ru; 2 Department of Therapy No. 7, Medical Center Hospital of the President’s Affairs Administration of the Republic of Kazakhstan, Astana 010000, Kazakhstan; naazgul@mail.ru; 3 Laboratory of Personalized Genomic Diagnostics, Medical Center Hospital of the President’s Affairs Administration of the Republic of Kazakhstan, Astana 010000, Kazakhstan; labgen-astana@inbox.ru; 4 Institution «National Medical Cardiology Research Center Named After Academician Ye. I. Chazov» of the Ministry of Health of the Russian Federation, Moscow 105064, Russia; trukhiniv@mail.ru; 5 National Scientific Shared Laboratory of Biotechnology, National Center of Biotechnology Limited Liability Partnership, Astana 010000, Kazakhstan; zholdybayeva@biocenter.kz

**Keywords:** idiopathic pulmonary arterial hypertension, BMPR2, genetics

## Abstract

Introduction: Idiopathic pulmonary arterial hypertension (IPAH) is a progressive and fatal disease. The aim of this study was to evaluate the association of polymorphism of the type 2 bone morphogenetic protein receptor gene (BMPR2) with the risk of IPAH development in an ethnic group of Kazakhs. We also describe the clinical and hemodynamic characteristics and outcomes of patients with and without carriers of BMPR2 gene mutations in IPAH. No available research highlights this problem in an ethnic group of Kazakhs. Materials and methods: A total of 53 patients of only Kazakh nationality with IPAH participated in the study. Clinical, functional, and hemodynamic characteristics, as well as the outcome of the disease, were compared among carriers and non-carriers of the BMPR2 mutation. Results: When receiving IPAH diagnosis, the average age of patients was 40.0 (32.0–48.0) years. Women predominated among the patients (86.8%). Of these, 17 (32.0%) were carriers of the gene mutation, and 36 (68.0%) did not have this mutation. The results of our research demonstrate that the Rs17199249 variant in BMPR2 contributed to increased susceptibility to IPAH. The T allele was associated with an increased risk of IPAH, with T = 75 (70.75%), G = 31 (29.24%), MAF—0.2925, x^2^—0.001, and HWE *p*—0.975. Carriers of the BMPR2 mutation were predominantly women (80.0%), and they had higher pulmonary vascular resistance (8.7–14.9 vs. 5.9–12.6 WU; *p* = 0.038), a low cardiac index (1.9–2.6 vs. 2.3–3.1 L/min per m^2^; *p* = 0.027), and a shorter time to death (*p* = 0.022). Conclusions: This is the first study of the genetic causes of IPAH that demonstrates the genetic polymorphism of BMPR2 is associated with an increased risk of IPAH developing with worse hemodynamic parameters and clinical outcomes.

## 1. Introduction

Pulmonary arterial hypertension (PAH) is a rare disease associated with unfavorable prognosis of a pressure increase in the small circle of blood circulation. The phenotypes of PAH are diverse and include idiopathic PAH (IPAH), the cause of which is unknown, and hereditary PAH (HPAH), which is associated with genetic changes or illustrates familial aggregation [1,2].

IPAH is a sporadic disease without any family PAH history or known causes. IPAH prevalence is 5.9 cases per 1,000,000 of the population. HPAH is diagnosed if there is a positive family history or a pathogenic variant is detected. IPAH is the most common PAH form according to the registers of European countries (France, Great Britain, and Ireland), the United States of America, and the Russian Federation [3]. PAH occurs in 15–50 patients per million of the population in Europe and the United States, of which hereditary, idiopathic, and anorexigen-induced PAH is 52.6% [4]. According to the Russian Register, these variants account for 41.5% of all PAH forms [5,6]. There is currently no register of patients with PAH in the territory of the Republic of Kazakhstan. Some highly specialized centers create databases, but there is no unified system for registering patients with PAH.

Currently, IPAH is increasingly considered a multifaceted disease, including many points of interaction between genetics, metabolomics, imbalance of vasoconstrictor and vasodilator reactions, endothelial and smooth muscle dysfunction, and more. Available genetic studies of HPAH and IPAH in individual populations have shown that the superfamily of transforming growth factor-β (TGF-β) plays an important role. At the same time, the identified mutations occur in the receptor of the type 2 bone morphogenetic protein (BMPR2), type 1 activin receptor-like kinase (ALK1), endoglin, and SMAD9 [7]. The BMPR2 mutation has been identified as the main genetic cause of PAH. It accounts for 75–90% of cases of HPAH and 3.5–40% of sporadic cases with an autosomal dominant type of inheritance [8,9]. BMPR2 deficiency leads to abnormal overactivation of the TGF-β signaling pathway, which causes excessive proliferation of vascular smooth muscle cells in pulmonary arterioles [10,11,12,13,14]. Patients with PAH and BMPR2 genetic variants tend to have the earlier onset and the worse clinical scenario of the disease course. The international consortium centrally compared and coordinated the data of 1550 patients with PAH from eight cohorts of six countries. The results of the study show that when identifying the BMPR2 mutation, PAH manifests at a younger age, has a more severe course, and has an increased risk of death or lung transplantation compared to patients without the BMPR2 mutation [15]. It is well known that patients with positive BMPR2 mutation have worse hemodynamic parameters at admission and worse disease outcomes compared to patients diagnosed with PAH without mutations [16]. In addition, a total of 17 PAH genes were acknowledged at the Sixth World Symposium on Pulmonary Hypertension. Many of these genes have been shown to belong to or be associated with the BMPR2/transforming growth factor (TGF)-β pathway [11,13,17].

Apart from BMPR2 pathway genes, disease-causing variants in genes strongly associated with PAH have also been identified in genes that encode a plasma membrane protein (caveolin-1 (*CAV1*)), a potassium channel protein (*KCNK3*), and an ion channel protein (*ATP13A3*) [18]. For *KCNK3*, *ATP13A3*, and *GDF2*, not only monoallelic but also biallelic variants have been described with a more severe presentation of the respective patients [19,20]. Genetic screening to detect patients with mutations and then identify close relatives is an important aspect. As noted in previous reports, annual monitoring of these asymptomatic individuals can provide earlier PAH diagnosis and earlier interventions that could potentially affect morbidity and/or mortality [21]. For example, in 2020, researchers of the United States Pulmonary Hypertension Scientific Registry (USPHSR) provided data from the first PAH patient registry in the United States, including genetic information. Genetic testing identified pathogenic or suspected pathogenic variants in 67 out of 499 (13%) participants and reclassified 40 out of 218 (18%) patients diagnosed with IPAH and 13 out of 256 (5%) patients diagnosed with associated PAH (APAH) to HPAH [22]. The detection of *BMPR2* mutations is a crucial step in the genetic diagnosis of PAH. This is very important for personalized therapy and genetic counseling [23,24,25].

## 2. Materials and Methods

**Population study.** The study included 53 patients of the Kazakh ethnic group with IPAH examined at the specialized National Scientific Cardiac Surgery Center (Astana, Kazakhstan) in the period from 2016 to 2022. All patients underwent thorough clinical examination, which made it possible to exclude other known causes of PAH. For the final verification of the clinical diagnosis of IPAH, all examined patients underwent catheterization of the right of the heart (the protocol of diagnosis and treatment in the Republic of Kazakhstan, as well as according to the recommendations of the European Society of Cardiology (ESC)).

A total of 53 examined patients with IPAH were identified as the main group, which consisted of an ethnic group of Kazakh nationality. The control group included 125 practically healthy individuals, comparable in age and gender and without a family history of PAH or other cardiovascular diseases (congenital heart defects, coronary heart disease, chronic heart failure, or arterial hypertension).

The demographic and clinical characteristics of all the surveyed were studied. Informed consent regarding the purpose and procedure of this study was obtained from each patient. To analyze the association between BMPR2 and the risk of IPAH, a stratified assessment was performed depending on hemodynamic parameters: mean right atrium pressure (mRAP), mean pulmonary arterial pressure (mPAP), the Fick cardiac index (CI), pulmonary vascular resistance (PVR), and the systemic vascular resistance index (SVR I). The scientific study was approved by the Ethics Committee of Karaganda Medical University NC JSC (Protocol No. 62, dated 12 April 2021).

**Single nucleotide polymorphism (SNP) choice and genotyping.** The choice of SNP was based on a literature review, allele frequency, and functional position. The study material was venous blood of patients with IPAH and practically healthy individuals. Genomic DNA was isolated from whole blood anticoagulated with ethylenediaminetetraacetic acid (EDTA) using PureLink^®^ Genomic DNA Kits (K1820-02) (Thermo Fisher Scientific, Waltham, MA, USA) in accordance with the protocol recommended by the manufacturer. Flanking primers and destructible fluorescent probes were developed to analyze the single nucleotide polymorphism of the BMPR2 (rs1061157), BMPR2 (rs2228545), BMPR2 (rs17199249), and BMPR2 (rs113305949) gene loci. Table 1 shows the sequences of primers and probes.

**Genotyping.** Real-time PCR was performed in an amplifier CFX96 (BioRad, Hercules, CA, USA). The amplification program for the thermal cycler had the following temperature conditions: +95 °C (3 min)—1 cycle; +95 °C (10 s), +60 °C (40 s, optical detection)—45 cycles. The results were analyzed in the Laboratory of Personalized Genomic Diagnostics, Medical Center Hospital of the President’s Affairs Administration of the Republic of Kazakhstan. After automatic annotation and visual inspection, genotypes were determined for each sample (patient).

## 3. Statistical Analysis

Statistical analysis was performed with the use of R version 4.2.1 (R Foundation for Statistical Computing, Vienna, Austria, 21 March 2022). Descriptive statistics were calculated and presented as means (*M*) and standard deviations (*SD*) for continuous data with symmetric distribution, while medians (*Mdns*) and interquartile ranges (*IQRs*) were displayed otherwise. Frequencies (*n*) and percentages (%) were presented for categorical data. Distribution of the data was evaluated by analyzing Skew and Kurtosis, as well as the visual inspection of histograms. Levene’s test was utilized to assess the assumption of homogeneity of variance. Univariate associations were studied with the unpaired samples *t*-test for continuous data following parametric assumptions; otherwise, the Mann–Whitney *U* test was applied. In the case of categorical data, Pearson’s Chi-Square test was employed.

Furthermore, Pearson’s Chi-Square test was utilized to assess the correspondence of the distribution of genotype frequencies to the Hardy–Weinberg equilibrium. The results are consistent with the Hardy–Weinberg law at *p* < 0.05.

Finally, associations of BMPR2 mutation status with risk of death were assessed using Cox proportional hazards regression models. Survival curves comparing patients with and without BMPR2 mutations were calculated using unadjusted Kaplan-Meier estimates and compared using the log-rank test.

The sample size was calculated using an online sample size calculator (https://www.calculator.net/sample, accessed on 3 April 2021). The sample size was sufficient to identify the association between the studied gene and IPAH (95% power, 3% disease prevalence, and 4.59% error).

The results were considered statistically significant at *p* < 0.05 throughout.

## 4. Results of the Study

**BMPR2 Mutations.** Table 2 shows the distribution of genotypes and alleles in the studied polymorphisms of the BMPR2 gene in the main group (IPAH) and the control group.

Table 2 demonstrates that rs2228545 in the BMPR2 gene did not correspond to the Hardy–Weinberg equilibrium and was excluded from subsequent analysis. The frequency of the minor A allele of the rs1061157 polymorphism (G > A) was 0.2353, while the MAF from the database (Global1000G) was A = 0.109175 (https://www.ncbi.nlm.nih.gov/snp/rs1061157, accessed on 5 April 2024). It should be noted that the frequency of occurrence of the A allele in the Kazakh population is higher than in Asian populations (A = 0.111). The frequency of the minor A allele of the rs113305949 polymorphism (C > A) was 0.1375, while the MAF from the database (Global1000G) was A = 0.0044 (https://www.ncbi.nlm.nih.gov/snp/rs1061157 accessed on 5 April 2024). It should be noted that the A allele is found in the Kazakh population, whereas in Asian populations, it is not (A = 0.000). The frequency of the minor T allele of the rs17199249 polymorphism (T > G) was 0.2925, while the MAF from the database (Global1000G) was G = 0.125138 (https://www.ncbi.nlm.nih.gov/snp/rs17199249 accessed on 5 April 2024). It should be noted that the frequency of occurrence of the T allele in the Kazakh population is similarly higher than in Asian populations (T = 0.0025). Compared with the G allele, the T allele can significantly increase the risk of IPAH developing (T = 70.75%, G = 29.24, MAF—0.2925, χ^2^—0.001, HWE *p*—0.975).

**Description of the cohort.** A total of 53 patients with IPAH participated in the study, of whom 17 (32.0%) were carriers of the gene mutation, and 36 (68.0%) did not have this mutation. The assessment of the association of the genotype with clinical and hemodynamic parameters reveals a statistically significant difference in PVR (*p* = 0.038) within the main group. Patients with IPAH who were carriers of the mutation (*n* = 17) had higher PVR values than non-carriers of this mutation (*n* = 36). In addition (Table 3), patients with the mutation had significantly lower CI (*p* = 0.027) and LVEF (*p* = 0.017).

It should be noted that there were no significant differences between the average age at the time of diagnosis (*p* = 0.221). However, there was a significant difference in the distribution by sex (*p* = 0.040). Thus, 80.0% of mutation carriers were women among patients with IPAH, while only 10.5% were men.

**Correlation of BMPR2 SNP with some hemodynamic characteristics.** Table 4 presents data on the bivariate association between BMPR2 and the hemodynamic characteristics of patients.

The results indicate the relationship between BMPR2 and hemodynamic parameters of patients. Thus, it was found that BMPR2 (rs17199249) was significantly associated with PVR (*p* = 0.035).

**Clinical outcomes.** All patients were monitored for 5 years, and there were no dropouts from the study. The following cardiovascular events were registered during the monitoring period: 20 patients died, and 12 of them were carriers of the BMPR2 mutation.

According to the results from comparing data obtained from patients with IPAH (carriers and non-carriers of the BMPR2 mutation), the hazard ratio (HR) was 2.896 (95% CI [1.165–7.065]; *p* = 0.022), which demonstrates a higher association with fatal events in patients with IPAH who were carriers of the BMPR2 mutation.

In the group of patients with IPAH (*n* = 53), females (*n* = 46) prevailed, 14 (30.4%) of whom were carriers of the gene mutation, and 32 (69.6%) did not have the mutation. A comparative assessment of carriers and non-carriers of the BMPR2 mutation among females showed that HR was 3.142 (95% CI [1.212–8.143]; *p* = 0.019), which determines the higher probability of death among female patients with IPAH associated with the mutation carrier. At the same time, among male patients with IPAH (Table 5), the HR was 1.414 (95% CI [0.088–22.64]), which was not statistically significant (*p* = 0.806).

**Figure 1:**
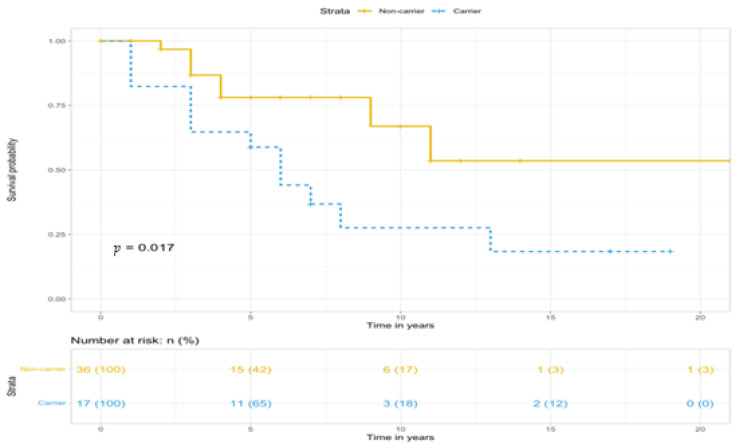
Kaplan–Meier survival curves according to BMPR2 mutation status. Total patients.

When comparing the survival curves of carriers and non-carriers of the gene mutation, it was found (Figure 1) that the survival rate of carriers is statistically significantly lower than in patients without the gene mutation (*p* = 0.017).

**Figure 2:**
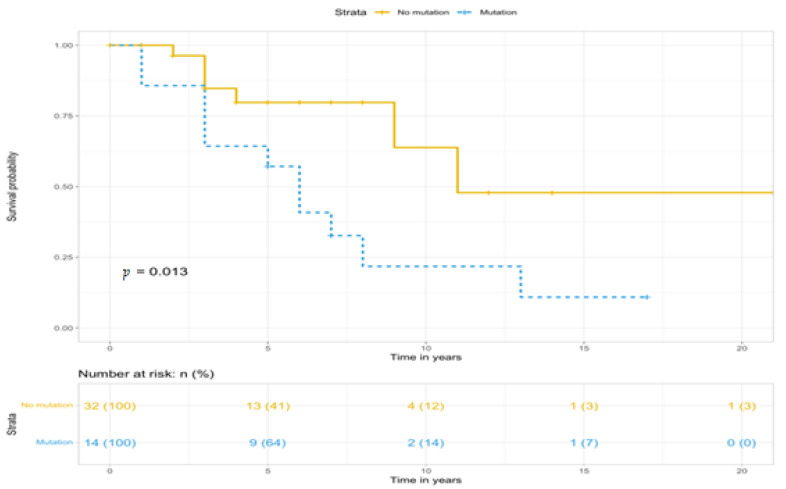
Kaplan–Meier survival curves according to BMPR2 mutation status among female patients.

The comparative assessment of the survival curves of carriers and non-carriers of the gene mutation among female patients shows (Figure 2) that the survival rate of female carriers of the gene mutation is statistically significantly lower than in patients without the gene mutation (*p* = 0.013).

## 5. Discussion

It follows from the study results that the average age of patients with IPAH in the ethnic Kazakh group at the moment of diagnosis determination was 40.0 (32.0–48.0) years. This fact corresponds to similar data from the last European and North American registries [26]. It should be noted that the age at the time of diagnosis among women and men was almost the same for carriers and non-carriers of the BMPR2 mutation. This study is the first to be conducted among the Kazakh population and demonstrates the relationship between the genotypic frequencies of BMPR2 SNP and IPAH debut. The results of the study show significant associations between the polymorphism of the BMPR2 gene (rs17199249) and the risk of IPAH development. This association is higher in the Kazakh population compared to Central Asians and Europeans. At the same time, BMPR2 polymorphisms (rs1061157 and rs113305949) in the main group (IPAH) showed no significant differences compared with the control group.

Evans et al. conducted a meta-analysis of 1550 patients with idiopathic, hereditary, and anorexigen-related PAH from eight cohorts and revealed that 29% of the examined persons had *BMPR2* gene mutations [16]. The decrease in gene expression was registered even in patients with PAH without *BMPR2* mutations [27]. Data from the study of an Asian cohort from Korea demonstrated that the prevalence of *BMPR2* variants in Korean patients with IPAH was 22% [28]. A Japanese group reported *BMPR2* variants in all four patients with HPAH (4/4 = 100%) and 12 (12/30 = 40%) with IPAH, and this index was higher than in Caucasian patients [29]. A study by colleagues from central Taiwan revealed the *BMPR2* gene mutation in 17.8% (8/45) of primary patients with PAH, which is slightly lower than the previously recorded prevalence in other cohorts. However, patients of the Taiwanese cohort with the *BMPR2* mutation went to a clinic at a younger age and had higher mPAP, higher PVR, and worse CI [30]. In addition, more severe hemodynamic disorders (low cardiac index and high total pulmonary resistance) were registered in the ethnic group of Kazakhs with IPAH associated with BMPR2 mutations, which coincide with previously published observations by other authors [31,32,33].

The comparative assessment of overall survival from the period of IPAH diagnosis substantiation allowed the establishment that there were significantly more fatal outcomes among carriers of the BMPR2 mutation. In addition, cardiovascular events occurred much earlier in female carriers of the BMPR2 mutation than in male carriers of this mutation. The obtained data are similar to the results of the study by G. Pousada et al. [34] and demonstrate that higher PVR values were registered in patients with BMPR2 mutations. The facts obtained in this study conclude that the patients of the Kazakh ethnic group with IPAH associated with BMPR2 mutations had a more severe disease course and worse prognosis compared with patients with IPAH without this genetic defect. At the same time, Pfarr et al. [17] found significant differences only at low PVR values. In this regard, they proposed that in the presence of an association with BMPR2 mutations, patients should be classified as patients with HPAH and must undergo segregation analysis of the hereditary nature of the disease.

One of the limitations of this study was the sampling size. This problem may be considered in future studies.

## 6. Conclusions

This study is the first to report on the genetic basis of IPAH in the Kazakh ethnic group and highlights the important contribution of genetic polymorphism to the development of IPAH.

It was established that in the ethnic group of Kazakhs, polymorphism of the BMPR2 gene is associated with the risk of IPAH development and a more expressed degree of hemodynamic disorders in the form of low cardiac output and high peripheral resistance. Female individuals with higher fatal cardiovascular events significantly predominate among patients with IPAH associated with the BMPR2 mutation. The obtained results indicate the need for further studies on a larger number of patients with IPAH in order to expand the spectrum of mutations in populations of ethnic groups.

## Figures and Tables

**Table 1:** Type 2 bone morphogenetic protein receptor primers.

Gene	Locus	Polymorphism	Primers	5′-3′
Bone morphogenetic protein receptor type II (*BMPR2*)	p13 chr 2	rs1061157	rs1061157-F	CCGAACTAATTCCAATAAC
			rs1061157-R	CTCCACTTACTCTGTATAC
			FAM-rs1061157-G	FAM-AGAGCACAGAGGCCTAATTCTC-BHQ1
			ROX-rs1061157-A	ROX-AGAAGAGCACAGAGACCTAATTCTC-BHQ2
*BMPR2*	P2 chr 37	rs2228545	rs2228545-F	CTGCATTGATTGTATTCATC
			rs2228545-R	TTCCCAAGAGACCTACTA
			FAM-rs2228545-C	FAM-AAGTTTGATTTGTGCTTGCTGCC-BHQ1
			ROX-rs2228545-T	ROX-CAAGTTTGATTTGTGCTTGTTGCCA-BHQ2
*BMPR2*	P2 chr 38	rs17199249	rs17199249-F	CCACGTTTTGTGTTTTATTG
			rs17199249-R	GGCAAGAGAACTAAGTGA
			FAM-rs17199249-T	FAM-CCCTTTTCTTTATTCAGCCCCTTA-BHQ1
			ROX-rs17199249-G	ROX-CCCTTTTCTTGATTCAGCCCCTT-BHQ2
*BMPR2*	P2 chr 38	rs113305949	rs113305949-F	TGACCTAAAACACTGTGA
			rs113305949-R	GTTGCTCACATATCAAAGA
			FAM-rs113305949-C	FAM-CATGCCAAGTCCCTATGAAGGAA-BHQ1
			ROX-rs113305949-A	ROX-CATGCCAAGTACCTATGAAGGAA-BHQ2

**Table 2:** Distribution of BMPR2 genotypes and alleles in IPAH and non-PAH groups.

BMPR2	Group	Genotype			Allele	MAF (Global1000G)	*x* ^2^	*HWE p*
		G/G	G/A	A/A				
rs1061157 (G > A)	IPAH (*n* = 51)	34 (63.8%)	10 (21.3%)	7 (14.9%)	G = 83 (81.37%), A = 19 (18.63%), MAF—0.2353	A = 0.11269/20,267	0.044	0.834
	Non-IPAH (*n* = 117)	64 (54.7%)	35 (29.9%)	18 (15.4%)				
		G/G	G/A	A/A				
rs2228545 (G > A	IPAH (*n* = 52)	3 (5.8%)	30 (57.7%)	19 (36.5%)	G = 51 (49.04%), A = 53(50.96%), MAF—0.6538	A = 0.033121/8480	12.517	<0.001
	Non-IPAH (*n* = 125)	7	102	16				
		T/T	T/G	G/G				
rs17199249	IPAH (*n* = 53)	30 (54.0%)	15 (30.0%)	8 (16.0%)	T = 75 (70.75%), G = 31 (29.24%), MAF—0.2925	G = 0.125136/38,507	0.001	0.975
	Non-IPAH (*n* = 125)	77 (61.6%)	29 (23.2%)	19 (15.2%)				
		C/C	C/A	A/A				
rs113305949	IPAH (*n* = 51)	39 (76.5%)	10 (19.6%)	2 (3.9%)	C = 88 (86.27), A = 14 (13.73) MAF—0.1375	A = 0.027411/846	0.002	0.962
	Non-IPAH (*n* = 124)	97 (78.2%)	22 (17.7%)	5 (4.1%)				

Note: Values are shown as number (*n*) and %. MAF—minor allele frequency. HWE—the Hardy–Weinberg equilibrium.

**Table 3:** Clinical characteristics of the study participants at the time of diagnosis.

Variable	All Patients (*n* = 53)	Patients with *BMPR2* Mutation (*n* = 17)	Patients Without *BMPR2* Mutation (*n* = 36)	*p*-Value
Age, years	45.0 (35.0–51.0)	46.0 (39.0–51.5)	44.0 (35.0–50.8)	0.333
Sex				
Female	46 (86.8%)	14 (82.4%)	32 (88.9%)	0.040 *
Male	7 (13.2%)	3 (17.6%)	4 (11.1%)	
Age at diagnosis IPAH, years	40.0 (32.0–48.0)	42.0 (35.5–49.5)	40.0 (28.5–45.0)	0.221
Family history of PAH				
Yes	47 (88.6%)	15 (100.0%)	32 (84.2%)	0.263
No	3 (5.7%)	0 (0.0%)	3 (7.9%)	
Unknown	3 (5.7%)	0 (0.0%)	3 (7.9%)	
6 MWD (6 min walk distance), m	328.26 (64.32)	333.53 (53.27)	326.18 (68.74)	0.712
NYHA				
I	4 (7.6%)	1 (6.7%)	3 (7.9%)	0.922
II	19 (35.8%)	6 (40.0%)	13 (34.2%)	
III	30 (56.6%)	8 (53.3%)	22 (57.9%)	
sPO2, %	95.0 (93.0–97.0)	95.0 (93.5–97.0)	95.0 (92.3–96.0)	0.564
Echocardiography				
LVEF, %	58.7 (57.0–63.0)	57.1 (56.0–58.2)	60.4 (57.8–64.0)	0.017 *
Pulmonary artery systolic pressure (PASP), mmHg	78.0 (66.0–90.0)	80.0 (63.5–92.0)	77.5 (66.0–89.5)	0.969
Early diastolic pulmonary regurgitation velocity, m/s	2.0 (1.9–2.7)	2.0 (1.9–2.4)	2.0 (1.8–2.8)	0.968
Fractional area contraction RV, (FAC), %	33.0 (30.0–35.0)	34.0 (31.5–35.5)	33.0 (29.3–34.0)	0.239
Tricuspid annulus plane excursion (TAPSE), mm	1.7 (1.6–1.9)	1.7 (1.6–1.8)	1.7 (1.6–1.9)	0.765
Right atrium area (end-systole), cm^2^	20.0 (17.0–26.0)	19.0 (18.0–25.5)	21.0 (17.0–26.0)	0.968
Pulmonary artery diameter, cm	2.8 (2.3–3.1)	2.6 (2.4–2.9)	2.8 (2.3–3.3)	0.313
Pleural effusion				
Yes	42 (79.2%)	14 (93.3%)	28 (73.7%)	0.122
No	11 (20.8%)	1 (6.7%)	10 (26.3%)	
Hemodynamic at diagnosis				
mRAP, mmHg	5.0 (4.0–6.0)	5.0 (4.0–6.0)	5.0 (4.0–6.0)	0.703
mPAP, mmHg	44.0 (36.0–52.0)	40.0 (37.0–51.0)	44.0 (36.3–51.3)	0.642
Fick CI, L/min per m^2^	2.3 (1.9–2.7)	2.2 (1.9–2.6)	2.6 (2.3–3.1)	0.027 *
PVR, WU	8.9 (6.0–13.6)	10.8 (8.7–14.9)	8.6 (5.9–12.6)	0.038 *
PVR I	15.0 (9.6–22.1)	16.2 (9.8–25.7)	13.9 (9.7–21.1)	0.413
SVR I	31.8 (25.6–36.4)	29.7 (26.0–34.6)	32.7 (24.2–36.6)	0.867
SvO_2_, %	67.3 (62.5–69.3)	66.3 (60.9–68.0)	67.5 (63.0–70.2)	0.291
Vasodilator responder				
Positive	27 (50.9%)	9 (60.0%)	18 (47.4%)	0.176
Negative	6 (11.3%)	3 (20.0%)	3 (7.9%)	
Not performed	20 (37.8%)	3 (20.0%)	17 (44.7%)	
Laboratories				
NT–proBNP, pg/mL	670.0 (258.0–1214.0)	670.0 (282.2–1031.0)	665.5 (237.0–1341.3)	0.992

Note: Results are presented as M (SD), Mdn (IQR), and *n* (%). * *p* < 0.05.

**Table 4:** Bivariate association between BMPR2 mRNA and patients’ hemodynamic characteristics.

	mRAP (mmHg)	mPAP (mmHg)	Fick CI (L/min per m^2^)	PVR (WU)	SVR (I)
rs1061157					
Mutation	4.0 (2.0; 5.0)	43.14 (15.67)	2.50 (2.30; 2.90)	9,40 (5.20; 13.95)	30.29 (8.28)
No mutation	5.0 (4.0; 6.0)	44.54 (11.54)	2.20 (1.90; 2.68)	8,85 (6.25; 13.01)	31.41 (8.31)
*p*	0.58	0.770	0.137	0.793	0.741
rs17199249					
Mutation	4.50 (3.75; 5.25)	38.25 (8.57)	2.58 (0.78)	5.90 (4.93; 9.33)	31.43 (8.79)
No mutation	5.0 (4.0; 6.0)	45.55 (11.99)	2.34 (0.53)	10.04 (7.25; 14.3)	31.60 (7.93)
*p*	0.470	0.107	0.277	0.035	0.956
rs113305949					
Mutation	5.0 (4.0; 6.0)	46.0 (2.83)	2.30 (2.05; 2.55)	6.60 (6.0; 7.20)	24.70 (19.65; 29.75)
No mutation	4.0 (4.0; 4.0)	44.36 (11.99)	2.30 (1.90; 2.68)	9.25 (6.25; 13.80)	32.15 (25.95; 36.64)
*p*	0.440	0.849	0.853	0.478	0.437

Note: Results are presented as M (SD) for symmetrical continuous data, Mdn (P25; P75) for non-symmetrical continuous data, and *n* (%) for categorical data.

**Table 5:** Hazard ratios of mortality associated with BMPR2 mutation in total, female, and male patients.

	BMPR2 Mutation		*p*-Value
	Carrier	Non-Carrier	
Total			
*n*	17	36	0.022
Deaths, *n* (%)	12 (70.6%)	8 (22.2%)	
HR (95%CI)	2.869 (1.165–7.065)	REF	
Female patients			
*n*	14	32	0.019
Deaths (*n*, %)	11 (78.6%)	7 (21.9%)	
HR (95%CI)	3.142 (1.212–8.143)	REF	
Male patients			
*n*	3	4	0.806
Deaths (*n*, %)	1 (33.3%)	1 (25%)	
HR (95%CI)	1.414 (0.088–22.64)	REF	

Note: HR—hazard ratio; 95%CI—95% confidence interval.

## Data Availability

The original contributions presented in this study are included in the article. Further inquiries can be directed to the corresponding author.
